# Re-expansion of Osteoporotic Compression Fractures Using Bilateral SpineJack Implants: Early Clinical Experience and Biomechanical Considerations

**DOI:** 10.7759/cureus.4572

**Published:** 2019-04-30

**Authors:** Robert E Jacobson, Anastas Nenov, Hoang D Duong

**Affiliations:** 1 Neurological Surgery, University of Miami Hospital, Miami, USA; 2 Interventional Radiology, Memorial Healthcare System, Hollywood, USA; 3 Interventional Neuroradiology, Memorial Healthcare System, Hollywood, USA

**Keywords:** spinejack, osteoporotic vertebral fractures, vertebroplasty, kyphoplasty, thoraco-lumbar fractures, balloon kyphoplasty, adjacent level fractures, intradiscal pressure, restoration vertebral height

## Abstract

Thoraco-lumbar osteoporotic compression fractures have a higher incidence of continued collapse with development of deformity and progression to vertebra plana when untreated and even after vertebral augmentation (VA) or balloon kyphoplasty (BKP). Even when there is the restoration of height and improvement in angulation, multiple long-term follow-up series have repeatedly documented that over time, many patients lose the initial height correction and in a smaller group the vertebral body re-collapses leading to the development of progressive deformity with an increased risk for adjacent level fractures. At first, larger balloons and more cement were used to try and avoid these problems, but it did not reduce the risk of adjacent fractures. Several procedures were developed to place various types of intervertebral implants combined with bone cement to maintain the initial height correction. Initial studies with these implants showed a reduction in adjacent level fractures but the systems did not proceed to market. The SpineJack^R ^(SJ) system (Stryker Corp, Kalamazoo, MI), consisting of bilateral expandable titanium implants supplemented with bone cement, was first used approximately 10 years ago in Europe and recently gained FDA approval in the United States. This system provides more symmetric and balanced lateral and anterior support and is effective with lesser amounts of bone cement compared to BKP. Follow-up studies have documented that there is equal or better pain control, with better long-term results based both on maintaining vertebral height restoration and deformity correction. Most importantly, statistically it clearly reduces the risk of adjacent level fractures by at least 60%. The biomechanical effects of intravertebral implants for osteoporotic fractures in regard to the risk of adjacent level fractures and preliminary experience with the use of the SJ^ ^is reviewed.

## Introduction

Evolution of vertebral augmentation and kyphoplasty procedures**

Vertebroplasty (VP), also known as vertebral augmentation (VA) and balloon kyphoplasty (BKP), have been used for over 30 years for the treatment of osteoporotic vertebral compression fractures for both persistent pain after conservative treatment and correction of vertebral collapse and deformity. Deformity and the development of adjacent level fractures at and above these osteoporotic fractures are significant long-term problems seen commonly at the thoraco-lumbar junction [1-2]. Anatomic studies show that osteoporotic vertebral compression fractures are essentially comminuted 'eggshell' like fractures of the vertebrae initially affecting the cortical endplates with progression to various degrees of compression and collapse of the vertebral body [2]. Many fractures also have vertebral clefts that can be identified on magnetic resonance imaging (MRI) or computerized tomography (CT) that are regarded as a clear sign of micro-instability and if the cleft is not filled with cement the fracture can continue to progressively collapse [3-4]. VA without balloon cavity formation works because it allows the cement to flow along the fracture lines but generally cannot restore vertebral height or correct angulation [2,4-5]. The evolution of using unilateral or bilateral balloon expansion followed by cement injection, known as balloon kyphoplasty (BKP), compared to simple vertebroplasty, or vertebral augmentation (VA), was to better restore vertebral height and correct associated kyphotic deformity [2,4]. Clinical and radiologic observations found that vertebral fractures, especially with concurrent vertebral fluid filled clefts found on CT and MRI scans, often re-expanded when the spine was placed in extension, indicating micro-instability but also indicating the fracture height could be restored by re-expansion with a balloon [3-4]. However, fractures with these vertebral clefts are also more prone to progressive deformity and collapse [4]. Technically, the balloon is used to dilate and expand the osteoporotic fractured area but is then removed. The comminuted vertebral fracture is pushed around the balloon and this 'dilated' area is then filled with polymethylmethacrylate (PMMA) bone cement to try and maintain the height correction [2]. Balloons vary in size from 10.0 mm x 2.00 mm to 20.00 mm x 6.00 mm and can be straight or curved, all in an attempt to make larger cavities to better expand the vertebra and subsequently restore vertebral height and maintain this height correction by filling the cavities with cement [1-2]. However, creating one large central cavity or two bilateral cavities through balloon expansion dramatically alters the biomechanics of a comminuted osteoporotic compression fracture [6]. By creating large solid cement-filled solid structures within the fractured vertebral body, which then create different sheer and compressive stress lines within the fractured body as well as secondary effects on the adjacent osteoporotic vertebral body, the forces on the adjacent osteoporotic vertebra are changed [6-7]. Multiple large patient follow-up studies have shown two significant problems in both short- and long-term follow-up of BKP: loss of the initial height correction with subsequent angulation and an increased incidence of fractures, mostly at the superior adjacent vertebra [7-9]. Follow-up studies show that as early as one-month post-procedure, loss of height restoration, and recurrent collapse, leading to further sagittal kyphosis with angulation of the fractured vertebra is found in 5% to 12% of treated one level fractures [8-9]. These fractures are almost always found in their early stages in the anterior and inferior part of the superior vertebra indicating a reproducible biomechanical stress point [4-7]. Using follow-up CT scans, fractures at the adjacent vertebra without any identified subsequent injury, usually are found just above the BKP, in up to 25% of patients that later develop adjacent fractures within the initial 90 days after the first procedure, although many of these fractures are asymptomatic. Follow-up MRI scans have also identified early edematous change without collapse in the anterior-inferior adjacent vertebra even before a symptomatic collapse is seen [5,7,9]. Numerous factors are thought to contribute to this increased incidence of adjacent fractures, but the main theories include the severity of the underlying osteoporosis, anterior shifting of the center of gravity secondary to angulation of the fractured vertebra, as well as spinal angulation with kyphosis and the biomechanical load effects of large amounts of denser bone cement injected into the treated vertebra and its secondary effects on adjacent softer more osteoporotic vertebrae [10-11]. The shifting of the center of gravity more anteriorly has been considered a major biomechanical reason for these fractures but studies comparing VA to BKP have consistently shown a higher incidence of adjacent level fractures with BKP, which is thought to be related to the larger volume, solid cement filled defects created after balloon expansion which puts upward stress on the adjacent osteoporotic vertebra [6-7,10,12]. Series where larger amounts of cement were used consistently had an increased incidence of adjacent fractures [4-6]. Observations of patients with a non-fractured vertebra between two cemented fractures also have a higher fracture rate while fractures treated with cement, plus two level fixation above and below the cemented vertebra rarely fracture since the downward weight load is spread over multiple vertebrae and any kyphosis, and subsequent anterior shift of center of gravity, is corrected or limited in its progression with instrumented fixation above and below the fracture [12-13].

Rationale for using implantable support for treatment of osteoporotic compression fractures 

In efforts to maintain the height correction of treated fractures, initially 1-mm insertable polymethylmethacrylate (PEEK), stackable wafers and then expandable PEEK spirals supplemented by PMMA were tried. In each case, either by adding wafers in 1-mm increments or enlarging the spiral the height could be gradually corrected and then maintained by adding PMMA cement. In both examples, the correction was better maintained but most importantly, with both systems, clinical and radiologic follow-up demonstrated that there were less adjacent fractures compared to BKP [14-15]. However, each system had technical difficulties that restricted their ultimate use and marketability. The SJ system, originally developed in Europe, with over a 10-year follow-up, is a further advance of the implantable concept, using two expandable aligned, specifically sized titanium implants. Each implant extends in front of and in line with the pedicle, providing lateral support. The two implants then extend to just off-center anteriorly, on each side of the midline, adding additional anterior support. By expanding the implant, it is possible to symmetrically actually 'jack' up the collapsed superior endplate, reducing the collapse and more consistently maintain the height correction than possible with BKP [10,16,17]. Using a bilateral system, the fractured vertebra gets significant internal mechanical support both laterally and anteriorly, which is the maximum point of collapse and later wedge deformity. Follow-up studies directly comparing SJ to BKP as well as multi-center European studies of the SJ implants over 10 years demonstrated that the initial height correction obtained with SJ is maintained, especially in the center of the endplate fracture, is associated with less spinal deformity, less cement is used than with BKP and the incidence of adjacent fractures is significantly less, 3% to 5% compared to 15% to 20% with BKP [10,16,17]. 

## Technical report

**Figure 1:**
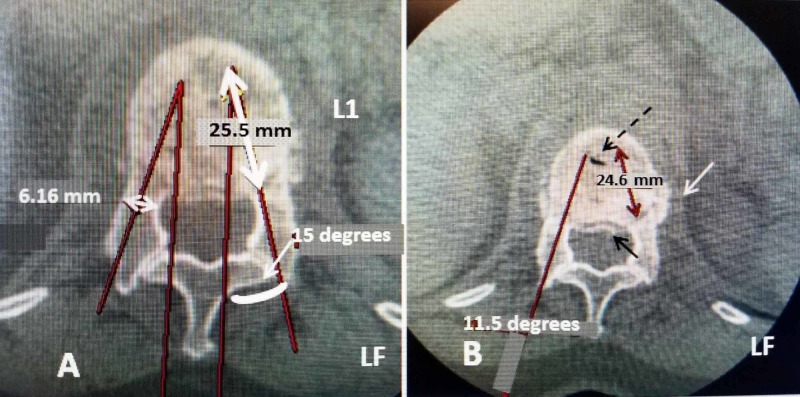
Measuring the fractured vertebral body to determine pedicle diameter and length to size correct implant to be inserted A: Axial CT scan of L1 fracture. The red line through the pedicle shows the angle (15 degrees) and position for placement of the implant. The 25.5-mm measurement is the maximum length of the implant (double-headed white arrow) on left (LF). The pedicle diameter 6.16 mm is also marked (small double-headed arrow on right over pedicle) to get the width of the closed implant that can be inserted. The final implant size is determined by both pedicle diameter (between the side cortex) and length to properly center the implants under the fractured endplates. In this case, a 5.8 x 20-mm implant can be used. B: Axial slice showing the angle (11.5 degrees) of insertion of the implant (solid red line). The fracture starts from the base of the L1 pedicle on the left side (LF) (solid white arrow). Minimal posterior displacement of the fracture into the spinal canal is seen (small solid black arrow). The length of the fractured endplate of 24.6 mm is measured (double-headed red arrow) which determines the maximum size implant that can be centered under the fracture. The slight lateral displacement of the fracture on the left can be seen (solid white arrow). A vertebral cleft is also identified in L1 in the anterior part of the fracture (dashed black arrow). The two SpineJack implants will be positioned anteriorly on each side of the cleft and cement will fill the cleft defect between the two implants.

The first step in using the implants is the measurement of the fractured vertebra for proper sizing and to make sure there is not extensive comminution of the pedicle or posterior vertebral wall [4,6-7]. Either computerized tomography (CT) or magnetic resonance imaging (MRI) films in sagittal, axial and coronal views are made to assess the integrity of the posterior vertebral wall, the pedicle and the depth of the anterior vertebra from the pedicle base and the diameter of the pedicle [10]. Precise measurements of the inner cortical pedicle size and the depth of the vertebra in a line projected from the anterior-medial to more posterior-lateral part of the vertebra will aid in planning where the implant will be positioned in line with the pedicle. This, in turn, allows exact sizing of the originally closed SJ so it can fit through the pedicle and is positioned properly to provide support along the maximum length of the fractured endplate and also centered underneath the fractured endplate (Figure 1).

**Figure 2:**
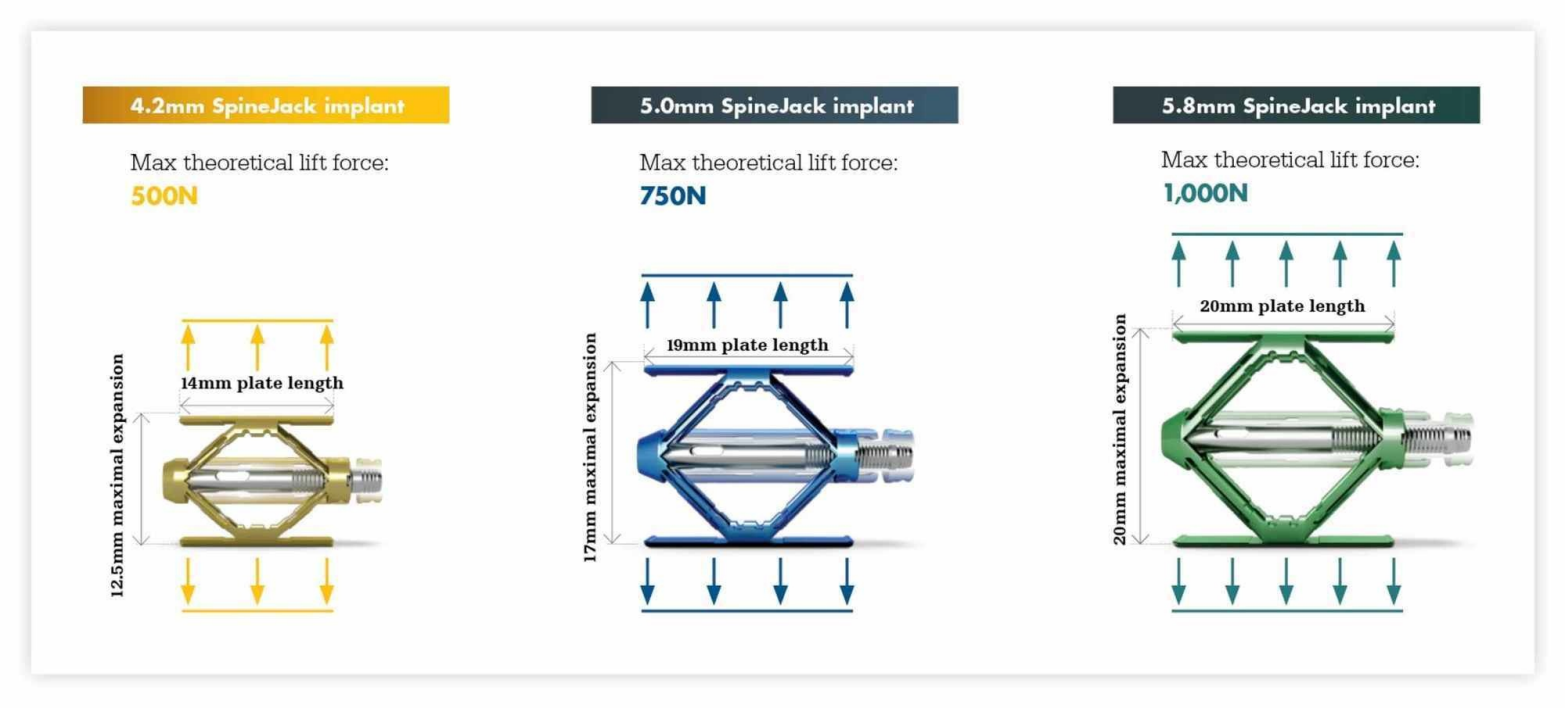
Dimensions of the different size SpineJack implants fully opened The implants come in three sizes: 4.2, 5.0 and 5.8 mm closed. The diameter of the closed implant is critical in determining which size implant can be inserted through the pedicle. When fully deployed and locked in place, they provide different dimensions and Newtons of load support, basically doubling in strength (500-1000 Newtons) from 4.2 to 5.8-mm-sized implants. The opened length of the struts that support the fractured endplate is shown for each implant, which also has different sagittal heights as indicated. (Picture Courtesy: Stryker Corp, Kalamazoo, MI)

The implants come in three different diameters for insertion into the pedicle with sizes of 4.2, 5.0 and 5.8 mm using the internal cortical diameter of the pedicle as the determining factor for size. When fully opened, each device has different heights and lengths, varying from 12.5 x 14.0 mm to 20.0 x 20.0 mm. The lift forces have been calculated and are reflected as compression load strengths described as force in newtons [10,16]. It is important to note the change in lift force with implant size, increasing 50% from 4.2 to 5.0 mm and 100% from 4.2 to 5.8 mm, so it may be important to use the largest size that will be fit through the pedicle. The 4.2-mm implant has been placed in mid and upper thoracic levels (Figure 2) [10].

**Figure 3:**
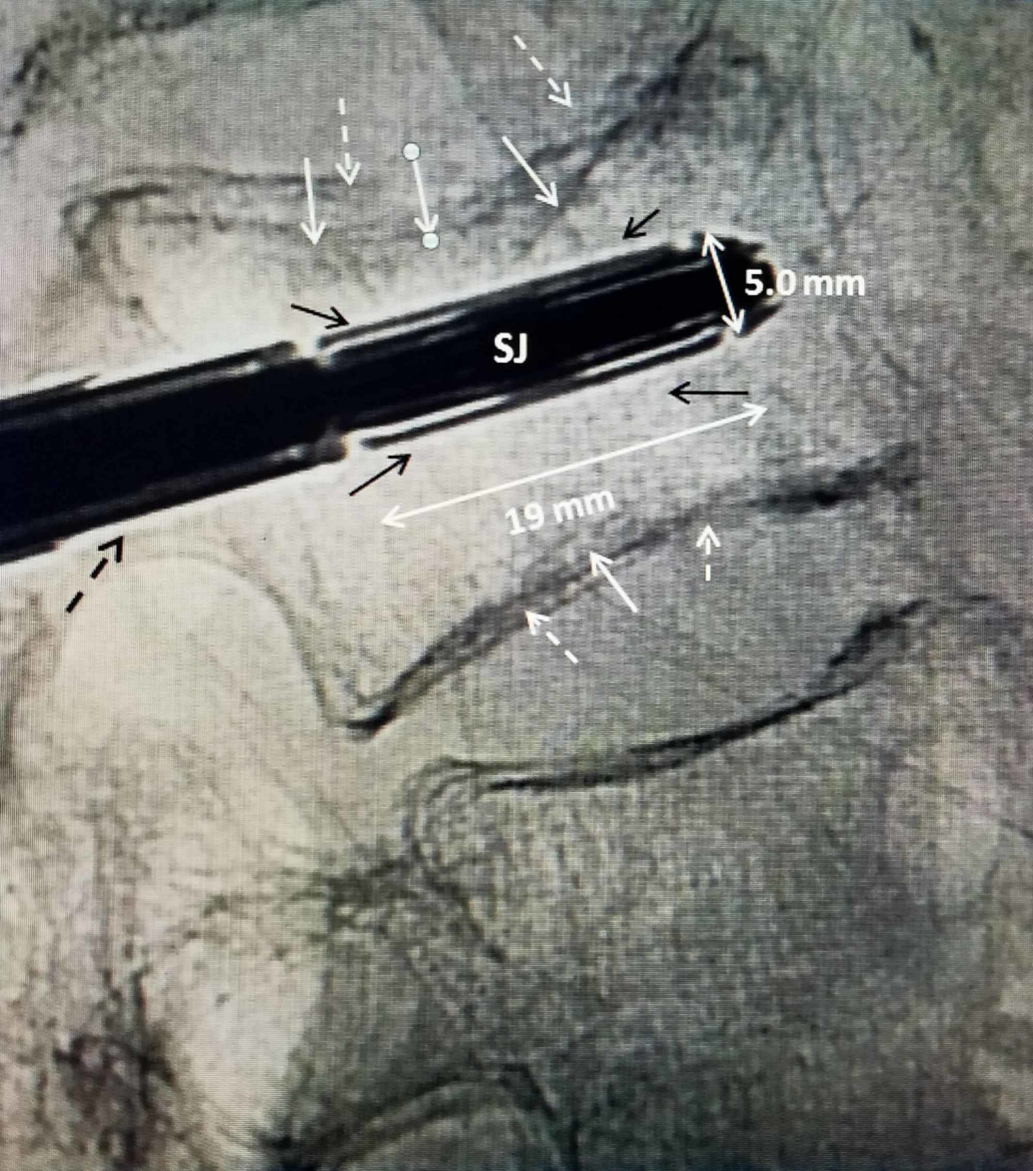
A closed 5.0-mm SpineJack implant aligned under superior endplate fracture The closed implant is centered directly under the compressed superior endplate fracture. The dimension of the closed SJ is marked, 19.0-mm length and 5.0-mm diameter (double-headed white arrows). The 'struts' of the implant are seen in a closed position (solid black arrows) and make part of the diameter of the closed implant. The double shadow of the compressed superior and inferior endplates from each side are seen (solid white arrows and dashed white arrows). The insertion handle of the SJ is seen going through the pedicle (dashed black arrow). SJ, SpineJack

Procedure: Under local anesthesia, similar to VA or BKP, with the patient placed in a prone position, after the fractured level is located, the device is inserted under local anesthesia. The fractured vertebra is localized and often if spinal hyper-extension is performed at this time, the collapsed vertebra can be seen to partially re-expand. This is found more commonly when CT or MRI reveals vacuum clefts within the fracture and is a sign of instability [3-4]. Next, two small 11-gauge vertebroplasty cannulas are used to access both pedicles but only up to the posterior margin of the vertebral body. A firm, 1.5-mm k-wire is then advanced to the anterior one-third of the vertebra on each side and the cannulas are removed. Next, a cannulated-sized reamer is used that matches the implant size selected. The reamer is passed over the k-wire and creates a path of the exact diameter as the closed implant for inserting the closed SJ device. The reamer also allows minor changes to correct angulation if needed. After both sides have been 'reamed', a sizer is used, that is exactly the same size as the closed implant so that the cavity can be properly positioned along the entire length of the fractured endplate. A lucent plastic marker with only a radio-opaque 5-mm tip is left in one side while the parallel opposite side is drilled so the two implants can be precisely aligned under fluoroscopic imaging (Figure 3).

**Figure 4:**
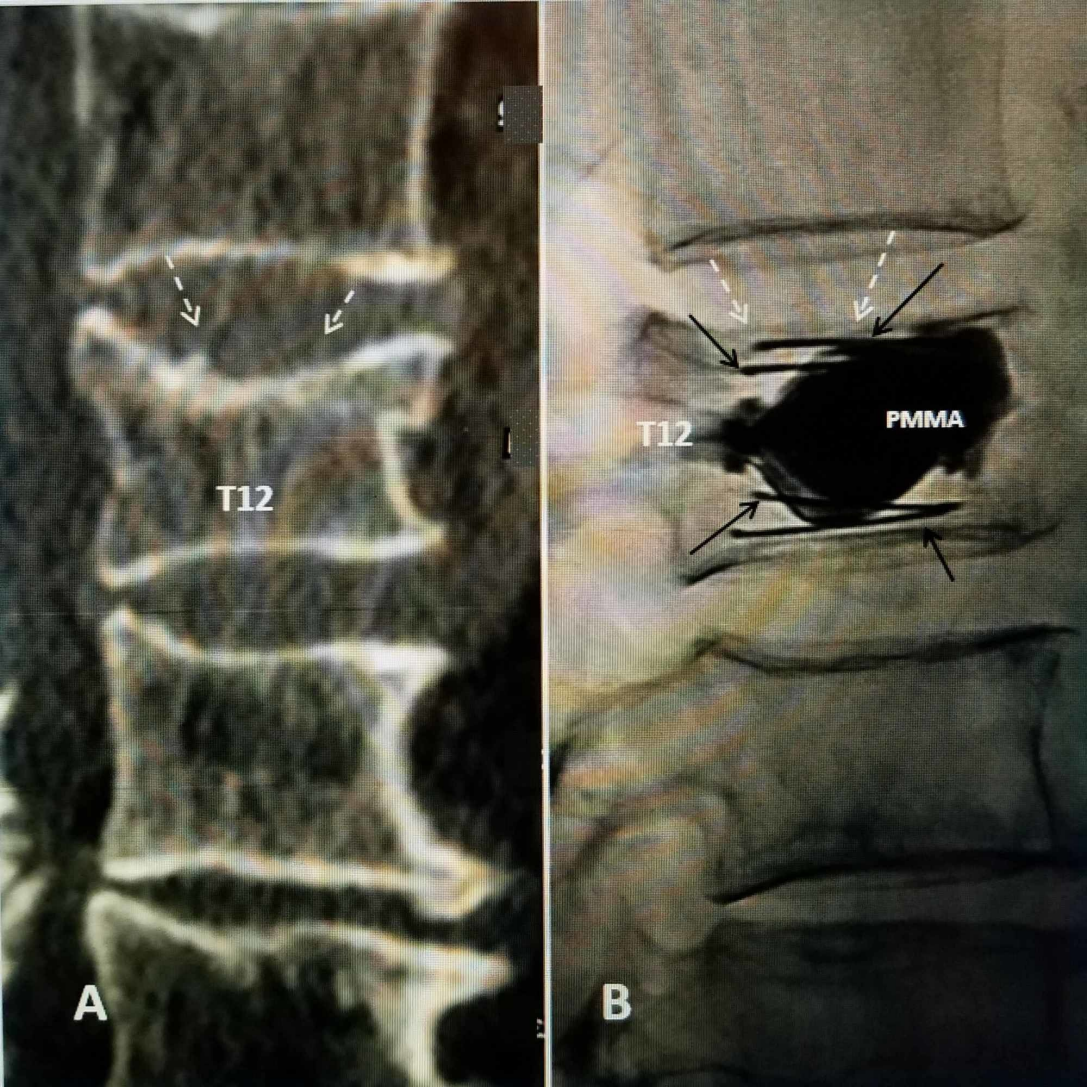
T12 fracture with two SpineJack implants placed in vertebral body with re-expansion of the superior endplate A: Pre-operative sagittal CT scan showing compression fracture of the superior endplate of T12 (dashed white arrows). B: Post-procedure digital fluoroscopic lateral image showing almost complete re-expansion of the superior endplate (dashed white arrows). The struts of the two parallel SpineJack 5.0-mm implants are clearly seen extending under the length of both superior and inferior endplates (solid black arrows). The cement is confined between the struts and the two implants (PMMA). PMMA, polymethylmethacrylate

The implant is then inserted on both sides and aligned superiorly and inferiorly, and then symmetrically expanded using a specially designed tool which locks into the device and expands the implant by pulling the two ends of the implant towards each other. Each implant is slowly expanded, alternating between implants so the expansion is smooth and symmetric without putting asymmetric stress on the fractured endplates on one side. This compression of the device causes the implant to open in an inferior-superior direction only, due to the machined grooves. A simple mechanism locks the implant into the desired position as controlled by the physician. Once the desired expansion is obtained, the device is left in place inside the restored vertebra and polymethylmethacrylate (PMMA) bone cement is injected (Figure 4). 

**Figure 5:**
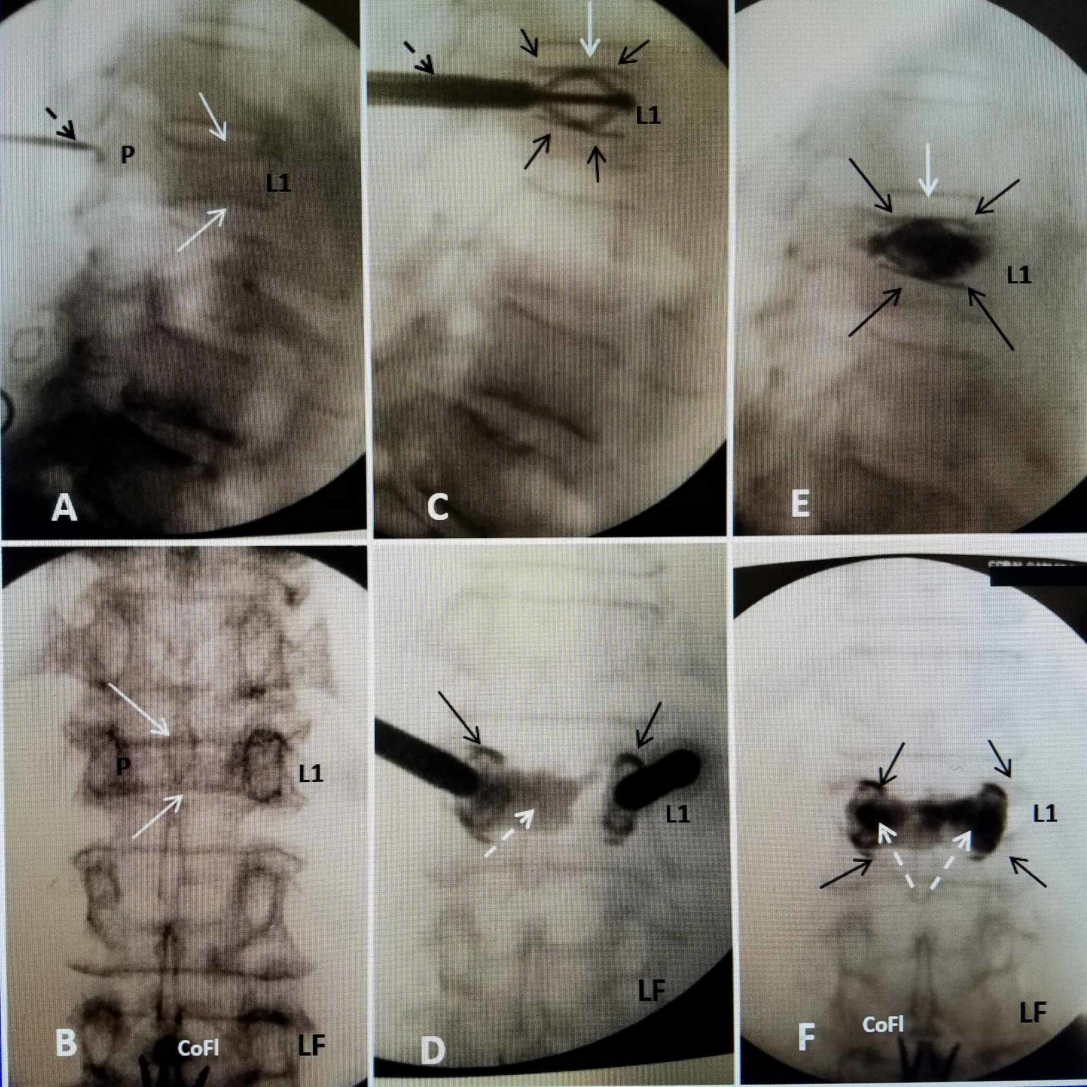
L1 acute compression fracture with sequential insertion of bilateral SpineJack implants followed by PMMA cement, demonstrating restoration of vertebral height A: Lateral intra-operative fluoroscopy showing superior endplate fracture of L1 (solid white arrow). The introducer cannula (dashed black arrow) for the k-wire is placed in line with the pedicle (P). B: Anterior-posterior (AP) film showing a biconcave collapse of both superior and inferior endplate of L1 (solid white arrows). C: Lateral fluoroscopy showing the introducer cannula (dashed black arrow). The expanded 5.8-mm SJ is clearly seen before bone cement injected. The two SpineJacks are parallel and the struts pass along the length of the endplates restoring vertebral height especially in the middle of the superior endplate (solid white arrow). D: Anterior-posterior (AP) intra-operative fluoroscopy showing the PMMA cement being injected unilaterally, on the right side, through the SJ implant (dashed white arrow). The struts are seen above and below the cement on both sides (solid black arrows). The cement is pooling around the device and across the midline (dashed white arrow). The implant on the left (LF) is expanded but has not been injected with cement yet. E: Lateral film after completed cement injection bilaterally. The superior and inferior struts (solid black arrows) appear to limit spread within and between the two implants. The fractured superior endplate has been restored to normal height (solid white arrow) with the struts supporting the superior endplate back to normal height. F: AP film after PMMA cement injected in both implants. The superior struts and inferior struts (solid black arrows) are seen bilaterally. Bone cement is filling both devices and completely across the midline (dashed white arrows), compared to what is seen in 'D'. PMMA, polymethylmethacrylate

Regular fluoroscopic controls throughout the operative procedure ensure correct implantation. The PMMA cement is injected through a pipette with a special attachment through the center mechanism of the device so cement exits small ports initially anteriorly and subsequently, the cement then spreads across the midline between the devices and spreads dorsally along and inside the opened implant. This minimizes the possibility of posterior cement spread and the risk of leakage of cement into the spinal canal. The general surgical time to perform bilateral insertion of SJ implants with PMMA cement is similar to bilateral balloon kyphoplasty. The resultant vertebra not only has height restoration but broad anterior and lateral support by the devices with cement intermingled in the titanium struts and across the midline (Figure 5).

## Discussion

In 2018, the SAKOS study (Prospective Comparative Study to Compare Safety and Effectiveness of Two Vertebral Compression Fracture Reduction Techniques) was finalized and the SJ device released under a 510K in the United States for painful vertebral compression fractures due to osteoporosis [10]. There have been several studies, including this latest randomized study where patients were similar age and pain severity, comparing 141 patients, 68 with SJ and 73 with BKP, over 12 months showed slightly better outcomes in pain scores over 12 months in the SJ group compared to BKP, as well as return to functionality but the restoration and maintenance of vertebral height was more marked, with a probability of 0.0035, combined with significantly less adjacent level fractures with no device failures or collapse. The restoration of height varied from one to eight mm but the average increase was primarily in the anterior and middle part of the collapsed vertebrae and was maintained in the SJ group both at six and 12 months. The BKP group also had a three-fold increase in the incidence of other thoracic fractures during the 12 months follow-up [10].

There are two key technical issues important in understanding the biomechanical advantage of the SJ in treating osteoporotic vertebral fractures and reducing the incidence of adjacent level fractures and deformity: first, broad strut like lateral and anterior support and second, correcting kyphotic angulation and maintaining height correction, which may effectively re-pressurize the superior adjacent disc that in turn adds support to the adjacent vertebrae [14-16]. In a longitudinal 12-month follow-up study after vertebral augmentation, 12% of patients developed adjacent level fractures with a mean time from the treatment of 68 days and 19% had further collapse of the treated vertebra in an average mean of 78 days. There are also case reports of the development of an adjacent level fracture as early as the first month after kyphoplasty [8]. The reported incidence of continued progression of a VCF at the same level of a previously treated fracture after vertebral augmentation is relatively small, ranging from 0.56% to 2% [7,11]. Several long-term studies post-vertebral augmentation show 10% to 15% gradual height loss in the treated vertebra in up to 30% of the patients with follow-up between 12 and 24 months [5,9,11]. It has been observed that even though the patient may be asymptomatic with regard to pain at the treated level, they can later present with adjacent vertebral fractures or progressive deformity. In a study with over a 12-month follow-up, it was found that even with the initial restoration of height and correction of kyphosis after balloon kyphoplasty, the correction is often lost over time, causing partial recurrent vertebral collapse and deformity [1,3-4,8]. Larger amounts of bone cement, especially if over 4 cc, and over correction of height plus fluid filled vertebral clefts are at least statistically related to an increased incidence of adjacent fractures [2,4,9,16]. Interestingly, the volume of cement used with the SJ has averaged only 2.5 cc around and between the two implants [10,17,18]. In patients with recurrent fractures in the same vertebra, the loss of vertebral height is much more acute and patients are usually symptomatic within the first 60 to 90 days post-procedure [7,9].

**Figure 6:**
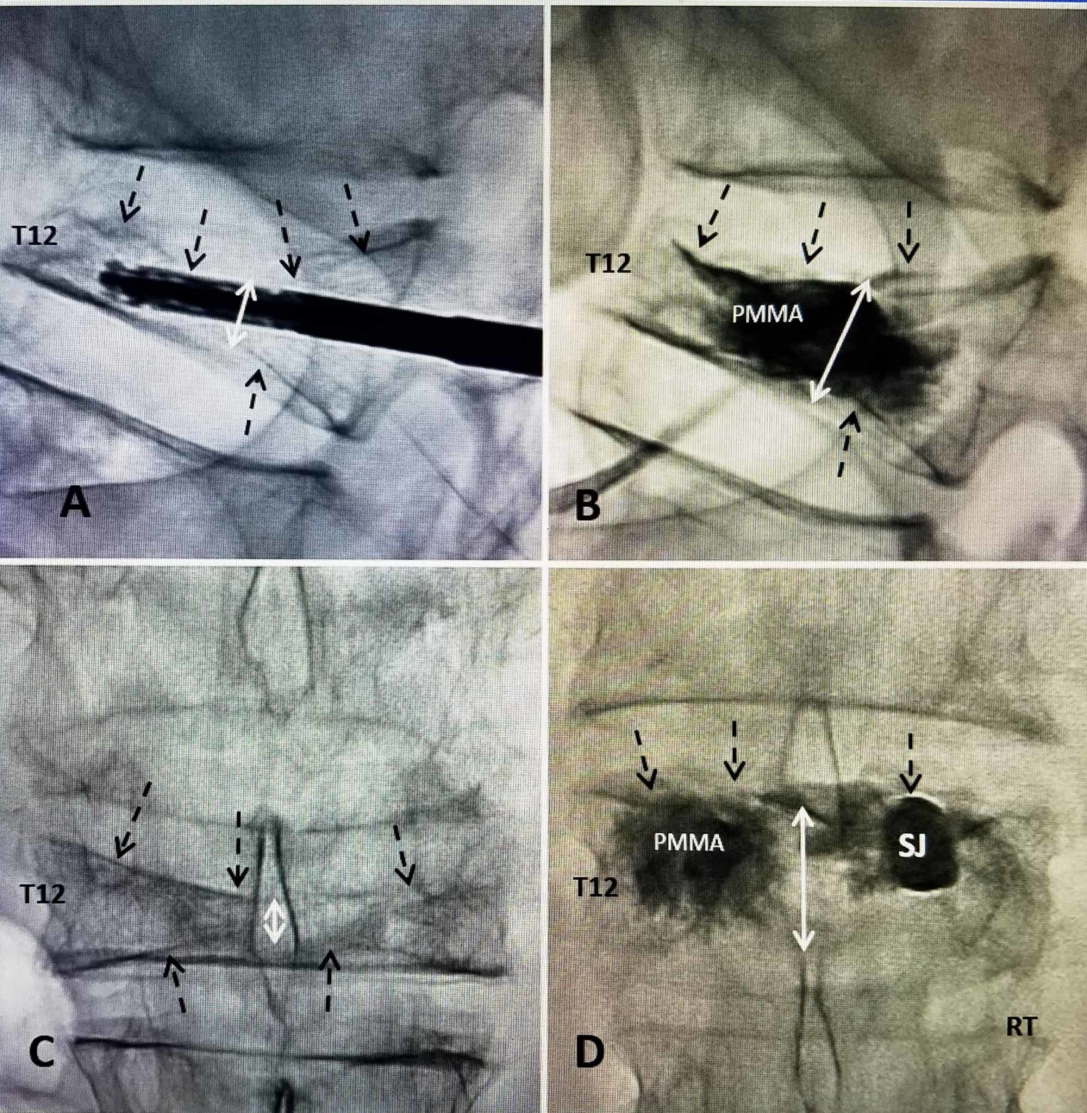
T12 acute compression fracture treated with 4.2-mm SpineJack A: Initial lateral intra-operative film showing a 70% middle (double-headed white arrow) and anterior compression fracture of T12. The closed 4.2-mm SJ and attached handle are seen centered under the fracture. The bi-concave superior endplate fracture (four dashed black arrows) and lesser and more posterior inferior endplate fracture is seen (single dashed black arrow). B: Lateral film after expansion of SJ in the center of T12 raising the middle part of the superior endplate fracture (three dashed black arrows). The 4.2-mm implant expands to 14.0 x 17.0 mm. It is filled with cement (PMMA). The middle T12 height has expanded three-fold (double-headed white arrow) and the inferior posterior endplate is also slightly expanded (single dashed black arrow). Note that the cement (PMMA) remains localized around the implant and none is tracking posteriorly toward the spinal canal. C: Anterior-posterior (AP) film before insertion of SJ showing marked collapse in the center of T12 (double-headed white arrow). The central vertebral collapse is mostly from depression of the center superior endplate (3 dashed black arrows) compared to the inferior endplate (two dashed black arrows). Normally the posterior and lateral walls remain closer to normal height with high degree collapse because of the stronger superior and medial pedicle cortex that maintains the posterior vertebral wall. D: AP fluoroscopy film after both SJ implants was inserted. Cement (PMMA) was injected in the left, before the right and is crossing the midline. The superior endplate is significantly elevated from the pre-implant film in 'C' and the midline height is restored (double-headed white arrow) especially in the center of T12 (three dashed black arrows). The right SJ has not been injected with cement.

Biomechanical studies found that a more trabecular spread of cement distributes the load and stiffness throughout a wider area of the fractured vertebra [2,7]. This may be one of the reasons for less adjacent fractures with simple VA in distinction to BKP, where the cement is used in larger volumes and is placed in a more globular concentration [6,9,11]. Cement in the region of the endplate nearest the adjacent fractured vertebra is also critical to reducing the incidence of continued collapse. In a study of technical reasons for the recurrence of a fracture within the same vertebra, it was found that one of the main reasons for the continued collapse of a previously treated vertebra and the adjacent vertebra was non-filling or insufficient cement placement around the fractured area and a lack of cement specifically filling near the fractured endplate [7,11]. The SpineJack^R ^implant^ ^creates a^ ^symmetric broader load support directly under the osteoporotic collapsing superior cortex and endplate based on precise placement of bilateral 'struts' which are supplemented by PMMA cement. The force generated by the bilateral implants varies by implant size but ranges from 500 to 1000 Newtons of load support under the fractured endplate, as well as load from the spinal column. (A Newton is equivalent to 4.48 pounds (lbs) and so 500 Newtons is 112 pounds per implant for the 4.2 mm SJ to 1000 Newtons for the 5.8 mm SJ or 224 lbs per implant). The PMMA cement is incorporated within the cancellous bone inside, around and especially between the two implants, both laterally and anteriorly creating a broad supporting ring under the endplate. The mechanical center of each 'strut' is under the fracture, the extended wings spread the load and provide a broad large force strength along the length of the fracture, similar to skis 'floating' above the snow, as the skier is centered on long extended skis [17-18]. These implants are effective with high degrees of vertebral collapse and angulation which is more common at the thoracolumbar junction (Figure 6).

The second biomechanical issue concerning vertebral fractures and their treatment relates to the observation of an increased incidence of developing a new fracture in the adjacent level above the fractured superior endplate with VA and even more frequently with BKP than is observed in the conservatively treated fracture [1-2,4,7]. The general bio-mechanical explanation for the higher observed incidence of these fractures has been the finding of increased stiffness of the original fractured vertebra injected with cement compared to the adjacent osteoporotic vertebra, further worsened by the use of larger volumes of cement in attempts to restore vertebral height [6]. When this is additionally combined with mechanical anterior shifting of the center of gravity of the sagittal spinal axis, there will be an increased load leading to more downward anterior pressure on the anterior and superior adjacent vertebra, leading to the next fracture in an already osteoporotic spine [8]. Yet in experimental cadaver studies, the superior adjacent vertebra above a BKP is subject to greater sheer forces and angulation then simple spinal compressive forces [6,8]. Several different groups recognized these bio-mechanical issues and sought to supplement the cement with different types of internal spinal vertebral implant systems that would maintain the height correction to avoid or minimize progressive collapse, possible recurrent fracture and subsequent spinal deformity [14-15]. Long-term SJ studies show a statistically significant reduction in the incidence of adjacent level fractures compared to no treatment, vertebroplasty and balloon kyphoplasty [10,14-16]. However, independent studies on intradiscal pressure demonstrate that there is significant reduction in pressure in the disc space above an untreated compression fracture which may lead to loss of mechanical support by the intervening disc between the already deformed fractured vertebra and the superior adjacent often osteoporotic vertebra [15,19]. Pressure measurements within the adjacent disc before and after expansion of the SJ support this theory, with post-implant measurements showing return to more normalized pressures [17,19-20]. This raises a possible additional bio-mechanical explanation for the significant reduction in adjacent level osteoporotic fractures as the restoration of disc pressure to normal provides better support to the adjacent vertebra and makes it less vulnerable to both compression and sheer forces [20].

## Conclusions

These examples of various fractures treated using SJ vertebral implants, combined with bone cement, demonstrate it is possible to not only restore vertebral height closer to normal but to obtain and maintain better correction because of the balanced implants. Long-term clinical experience with the SJ implant over 10 years, compared to balloon kyphoplasty, has demonstrated that these corrections are better maintained and associated with lower long-term spinal pain, and most importantly, significantly less regression of height correction and development of adjacent level fractures. Biomechanically, the broader support provided to the lateral and anterior parts of the fractured and depressed superior endplate minimizes the anterior shift of the center of gravity that is commonly seen with anterior fracture deformity and kyphosis. Several studies have demonstrated secondary effects on the supporting pressures within the superior adjacent disc above the fracture. This evidence supports the biomechanical concept that by restoring the disc pressure closer to normal, the intervening disc between the treated fracture and the superior adjacent vertebra adds support to the adjacent vertebra and the incidence of adjacent fractures is significantly decreased. 
